# Examination of unintended consequences of antibiotic use restrictions in food-producing animals: Sub-analysis of a systematic review

**DOI:** 10.1016/j.onehlt.2019.100095

**Published:** 2019-05-15

**Authors:** Karen L. Tang, Niamh P. Caffrey, Diego B. Nóbrega, Susan C. Cork, Paul E. Ronksley, Herman W. Barkema, Alicia J. Polachek, Heather Ganshorn, Nishan Sharma, James D. Kellner, Sylvia L. Checkley, William A. Ghali

**Affiliations:** a Department of Medicine, Cumming School of Medicine, University of Calgary, 3330 Hospital Drive NW, Calgary, Alberta T2N 4N1, Canada; b Department of Ecosystem and Public Health, Faculty of Veterinary Medicine, University of Calgary, 3280 Hospital Drive NW, Calgary, Alberta T2N 4Z6, Canada; c Department of Production Animal Health, Faculty of Veterinary Medicine, University of Calgary, 3280 Hospital Drive NW, Calgary, Alberta T2N 4Z6, Canada; d O'Brien Institute for Public Health, University of Calgary, 3280 Hospital Drive NW, Calgary, Alberta T2N 4Z6, Canada; e Department of Community Health Sciences, Cumming School of Medicine, University of Calgary, 3280 Hospital Drive NW, Calgary, Alberta T2N 4Z6, Canada; f W21C Research and Innovation Centre, Cumming School of Medicine, University of Calgary, 3280 Hospital Drive NW, Calgary, Alberta T2N 4Z6, Canada; g Libraries and Cultural Resources, University of Calgary, 2500 University Drive NW, Calgary, AB T2N 1N4, Canada; h Department of Pediatrics, Cumming School of Medicine, University of Calgary, 28 Oki Drive NW, Calgary, Alberta T3B 6A8, Canada; i Alberta Children's Hospital Research Institute, University of Calgary, 28 Oki Drive NW, Calgary, AB T3B 6A8, Canada; j Department of Microbiology, Immunology, and Infectious Disease, University of Calgary, 3330 Hospital Drive, NW, Calgary, AB T2N 4N1, Canada; k Alberta Provincial Laboratory for Public Health, Alberta Health Services, 3030 Hospital Drive, NW, Calgary, AB T2N 4W4, Canada

**Keywords:** Antimicrobial resistance, One health, Antimicrobial use

## Abstract

Antimicrobial resistance is considered one of the greatest threats to global and public health today. The World Health Organization, the Food and Agriculture Organization, and the World Organisation for Animal Health, known as the Tripartite Collaboration, have called for urgent action. We have previously published a systematic review of 181 studies, demonstrating that interventions that restrict antibiotic use in food-producing animals are associated with a reduction in antibiotic resistant bacterial isolates in both animals and humans. What remains unknown, however, are whether (and what) unintended consequences may arise from such interventions. We therefore undertook a sub-analysis of the original review to address this research question. A total of 47 studies described potential consequences of antibiotic restrictions. There were no consistent trends to suggest clear harm. There may be increased bacterial contamination of food products, the clinical significance of which remains unclear. There is a need for rigorous evaluation of the unintended consequences of antibiotic restrictions in human health, food availability, and economics, given their possible widespread implications.

## 1 Context

With increasing attention paid to the rapid rise in antimicrobial resistance and its resulting health and economic consequences, there is mounting pressure to develop strategies to promote prudent use of antibiotics in humans and in agriculture [1]. Though the World Health Organization (WHO) has made recommendations on prudent use of antimicrobials in food-producing animals as early as 1997 [2], they recently undertook a rigorous process, following international standards, to develop and publish formal guidelines on this topic [3]. These WHO Guidelines recommended both a reduction and restriction of antibiotics in food-producing animals, and were informed by our recent systematic review and meta-analysis showing that such measures likely reduce antibiotic resistance in animals and also in certain human populations (particularly those having direct contact with animals) [4]. Evidence though of potential unintended consequences is less clear. There are concerns that restrictions of antibiotic use in food-producing animals may negatively impact animal health and welfare, resulting in increased rates of infection and a paradoxical increase in antibiotic use for therapy [[5], [6], [7]]. Furthermore, antibiotic growth promoters have been used to maximize growth, production, and feed efficiency, resulting in some hesitation in response to complete bans of these products.[8] Increasing evidence suggests though, that the benefit of antibiotics for productivity is likely minimal in industrialized production,[[9], [10], [11]] with no significant long-term negative impacts seen when antibiotic growth promoters are eliminated.[[11], [12], [13], [14]].

McEwen et al. conducted a narrative review of 14 studies that examined unintended consequences of national-level restrictions of antibiotic use in food-producing animals [15]. Five studies reported no adverse consequences, while the others reported increases in certain diseases in the animals, increased antibiotic use for therapeutic purposes, and decreased feed efficiency. These effects tended to be small, temporary and likely to be mitigated by improved biosecurity, hygiene, and animal housing and husbandry practices. The authors concluded that the implementation of strategies to restrict antibiotic use in food-producing animals should not be delayed.

To add to this evidence base, we present here a sub-analysis of our previously published systematic review [4]. The methods have been described in detail in that publication. [4] In summary, we searched electronic databases Agricola (1970-present), AGRIS (http://agris.fao.org), BIOSIS Previews (1980-present), CAB Abstracts (1910-present), MEDLINE (1946-present), EMBASE (1974-present), Global Index Medicus (http://www.globalhealthlibrary.net; non-MEDLINE indices included AIM [AFRO], LILACS [AMRO/PAHO], IMEMR [EMRO], IMSEAR [SEARO], WPRIM [WPRO], WHOLIS [KMS], and SciELO), ProQuest Dissertations, and Science Citation Index (1899-present), in July 2016 with an update in January 2017. Inclusion criteria were original studies describing interventions to reduce antibiotic use in food-producing animals, and that compared proportions of antibiotic-resistant bacterial isolates in animals or humans between intervention and comparator groups. Any interventions that reduced or restricted one or more antibiotics, to any extent, were considered; these included mandatory or voluntary bans, antibiotic-free or organic production systems, national reduction targets, or requiring veterinary consultation or culture and sensitivity testing prior to antibiotic use. For this sub-analysis, we specifically identified the subset of studies that report unintended consequences of interventions that restrict antibiotic use in food-producing animals; the key findings from this sub-analysis are summarized below.

## 2 Findings

Of the 181 studies included in the original systematic review, 47 were included in this sub-analysis, on the basis of the studies explicitly reporting information on potential unintended consequences associated with antibiotic restriction strategies (Table 1). Detailed characteristics and quality assessments of the individual studies can be found in our prior publication [4]. The unintended consequence that was most frequently examined in this subset of studies was bacterial contamination and/or food safety. None explored adverse effects on human health or decrease in food availability for human consumption.

**Table 1:**
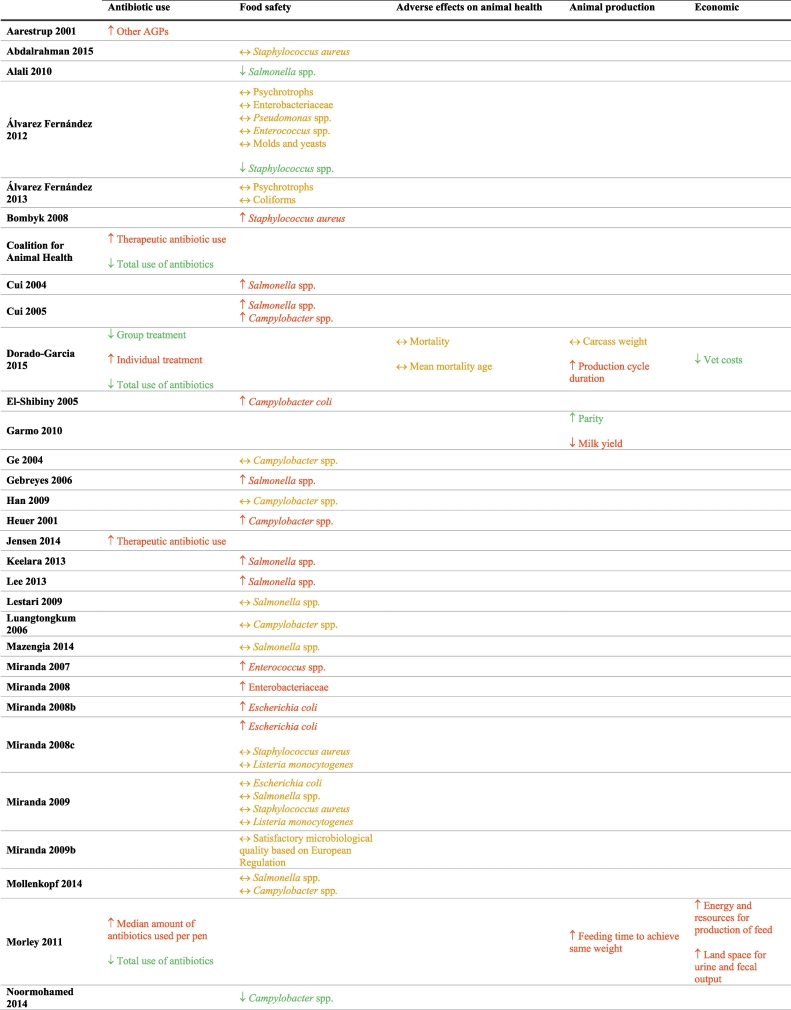

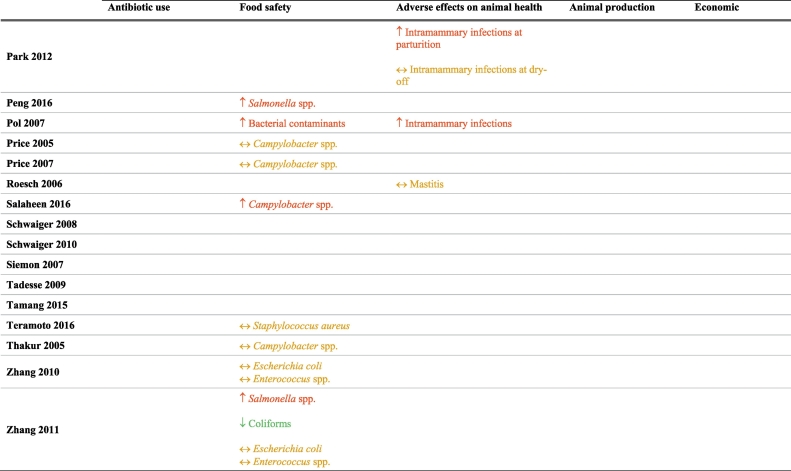
Unintended consequences of interventions restricting antibiotic use in food-producing animals.

Abbreviations: AGP – Antibiotic growth promoters; ↑ = increased in the intervention compared to the comparator group; ↓ = decreased in the intervention compared to the comparator group; and ↔ = no difference between the intervention and comparator groups.

Where Red = favors comparator group; Green = favors intervention group; Yellow = no difference between intervention and comparator group.

### 2.1 Antibiotic use (n = 5)

One study found an increase in the use of non-restricted antibiotic growth promoters (AGPs) after the ban of one specific AGP [16]. Four studies reported that though there was an increase in the use of therapeutic antibiotics to treat individual animals, there remained a reduction in the total amount of antibiotics used [5,[17], [18], [19]].

### 2.2 Food safety (n = 34)

Fifteen studies found an increased rate of bacterial contamination in retail meats when antibiotic restrictions were applied [[20], [21], [22], [23], [24], [25], [26], [27], [28], [29], [30], [31], [32], [33], [34]]. Eighteen studies reported either no difference in contamination rates or less contamination in the intervention group when the use of antibiotics was restricted [[35], [36], [37], [38], [39], [40], [41], [42], [43], [44], [45], [46], [47], [48], [49], [50], [51], [52]]. One study showed variable results depending on the bacteria in question [53].

### 2.3 Animal health (n = 4)

Two studies in dairy herds reported increased prevalence of intramammary infections and mastitis pathogens with restriction of antibiotic use (due to organic production) [33,54], while a third study showed no difference in mastitis between groups [55]. The single study that examined mortality reported no difference in either mortality rate or mean age at mortality in intervention versus comparator groups [17].

### 2.4 Animal production (n = 3)

Two studies reported adverse effects on animal production with increased feeding time to achieve target weight and increased production cycle duration [17,19]. One study showed variable results, with increased parity but lower milk yield in dairy cows [56]. The effects of antibiotic restrictions on animal production vary likely as they depend upon concurrent management changes implemented to promote animal health. For example, when Denmark banned antibiotic growth promoters, productivity improved likely due to a multimodal strategy that included increased veterinary oversight and changes to feed composition to include whole wheat and feeding enzymes.[14,57].

### 2.5 Costs and economics (n = 2)

One study estimated increased costs in animal production due to increased feeding time to reach target weight, when antibiotic use is restricted [19]. Another study reported decreased veterinary costs with antibiotic restriction; the specific cost inputs and drivers of this cost difference were not reported [17].

## 3 Interpretation of findings

This sub-analysis of our comprehensive systematic review suggests that unintended consequences are uncommonly reported in studies that are designed to examine the effect of antibiotic restrictions in food-producing animals on antibiotic resistance. Of the 181 studies included in our original systematic review, only 47 reported any unintended consequences. Of these, nearly one-third reported unintended consequences in the discussion section of the publication, without specifying these in a research question or objective.

Despite theoretical concerns that restrictions in antibiotic use in food-producing animals may result in numerous harms to both animal and human health, these are not borne out in our sub-analysis. The associations between unintended consequences and antibiotic restrictions are mixed across all outcome domains, with no clear or consistent trend. Half of the studies reporting on safety of retail food products suggest increased contamination when antibiotic restriction measures are in place. Because no study examined human health outcomes, the clinical significance of this is unclear.

We recognize that unintended consequences were not specifically the focus of our systematic review. As a result, this sub-analysis does not comprehensively capture all studies on this topic. Furthermore, all but two of the studies were undertaken in the United States of America or in Europe. Generalizability of our findings may therefore be limited, especially to low and lower-middle-income countries where management and hygiene practices may be less developed. However, our study complements the previously-mentioned paper on this topic by McEwen et al. [15], by virtue of our identification of a number of additional studies not covered by their recent review. Together, our two reviews provide value in summarizing an informative, though small, body of literature examining potential harms of interventions that restrict antibiotic use in food-producing animals. We demonstrate that future research on antibiotic restrictions in agriculture should more specifically consider their impact on unintended consequences. The increasing global efforts to reduce and restrict antibiotic use in food-producing animals present the perfect opportunity to conduct rigorous evaluations of potential harms and to provide insight regarding the role of local context in the relationship between antibiotic restriction and unintended consequences.

## Contributors

Each of the 12 authors meets the authorship requirements as established by the International Committee of Medical Journal Editors in the Uniform Requirements for Manuscripts Submitted to Biomedical Journals. All authors were involved in the design and development of the study. HG created the search strategy and conducted the literature search in electronic databases. DN conducted the grey literature search. KT and NC screened all studies for inclusion into the original systematic review and performed all study quality assessments. SC, PR, and HB provided input on studies where consensus could not be reached. KT, NC, DN, AP, and NS, performed data extraction. All authors contributed to data interpretation and data analysis. KT drafted the manuscript and all authors revised it critically for content. All authors have full access to all data and can take responsibility for the integrity of the data and accuracy of the data analysis. All authors have read and approved the manuscript.

## Role of the funding source

The WHO was involved in both the original systematic review and meta-analysis, as well as this sub-study. They were involved in developing the research question, the study design and the study protocol. They had no involvement in data extraction or interpretation of findings. The authors have been given permission by the WHO to publish this article. All had full access to all of the data and can take responsibility for the integrity of the data and the accuracy of the data analysis.
